# Assessing the reproducibility of Fourier-transform mid-infrared–derived biomarker clusters across lactations and their direct prediction from milk spectra

**DOI:** 10.3168/jdsc.2026-1060

**Published:** 2026-06-11

**Authors:** S. Franceschini, S. Czaplicki, N. Nickmilder, J. Leblois, C. Grelet, N. Gengler, H. Soyeurt

**Affiliations:** 1 TERRA Research and Teaching Centre, Gembloux Agro-Bio Tech, University of Liège, 5030 Gembloux, Belgium; 2 Department of Veterinary Management of Animal Resources, University of Liège, 4000 Liège, Belgium; 3 Walloon Breeders Association, 5590 Ciney, Belgium; 4 Walloon Agricultural Research Centre, 5030 Gembloux, Belgium

## Abstract

Fourier-transform mid-infrared (FT-MIR) spectroscopy is a powerful tool to predict fine milk composition but also indirect traits such as cow health at a large scale. Previously, an unsupervised learning approach applied to primiparous Holstein cows identified a cluster associated with negative energy balance and elevated concentrations of blood BHB and milk acetone, which are recognized indicators of metabolic disruptions. This study extended the methodology to multiparous cows, representing a broader variability. A hierarchical clustering pipeline was applied to over 1,712,417 records from primiparous and multiparous Holsteins, using 27 FT-MIR–based predictions, SCS, and milk yield. The approach was repeated 5 times for 4 different datasets: first, second, and third lactations, and a merged dataset for a total of 20 iterations. Across those iterations, the metabolic cluster was consistently found, and 2 partial least square discriminant analysis prediction models were estimated for each iteration using either the predictions or directly the spectra. The correlations between these predictions of all the models were between 0.82 and 1, suggesting that the models are similar. The cluster previously associated with udder health conditions was not consistently found across repetitions. To ensure transferability and facilitate the deployment of the results, a final model based on FT-MIR was estimated, presenting good performance with an accuracy in validation of 0.92. Those results prove that the metabolic cluster is extendable to other lactations and predictable directly from milk FT-MIR spectra.

## Highlights

• Clustering through a large database of FT-MIR predictions allows for pattern detection.
• Cow metabolic status has a strong signal in FT-MIR, which is consistent across lactations.
• Signal for mastitis is dependent on somatic cell count or somatic cell score and is not consistent across iterations.
• The cow metabolic status is predictable directly from the FT-MIR spectra.

## Graphical Abstract

**Figure:**
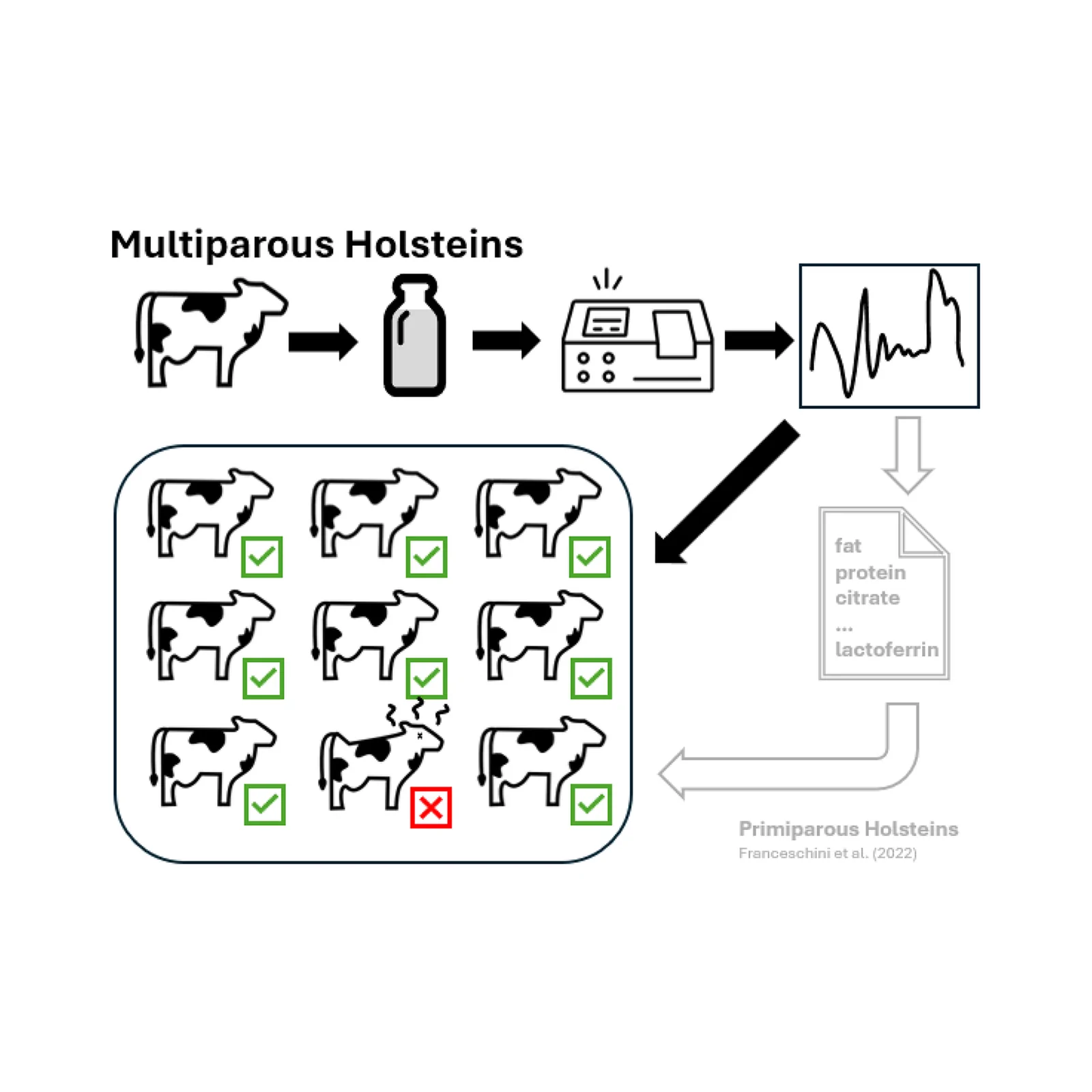
**Summary:** Fine milk composition, predicted through Fourier-transform mid-infrared (FT-MIR) spectroscopy for milk recording, is a powerful tool exploitable to detect cows with health issues. Building upon our earlier work conducted on primiparous Holstein cows, this study extends the use of an unsupervised learning method to multiparous cows while also estimating the robustness of the approach. The methodology developed was repeated 5 times across 3 different lactations. The correlations between the results for primiparous and multiparous cows confirm that the patterns detected are consistent and robust only for metabolic trouble detection. To facilitate practical deployment, a predictive model was developed to estimate cluster membership probabilities directly from milk FT-MIR spectra. The resulting model achieved a classification performance (accuracy = 0.92) comparable to that obtained in the previous study, reinforcing its potential for large-scale phenotyping and field application.

Disease events in dairy herds such as mastitis or ketosis can severely impair production and herd profitability while also compromising animal welfare. For instance, Puerto et al. (2021) showed that mastitis cows could lose between 382 and 989 kg in 305 DIM cumulative milk yield compared with healthy cows, and a systematic review from Cainzos et al. (2022) highlighted the considerable impact of ketosis across multiple countries with cost per case ranging between €19 and €812. Recent estimates indicate that common dairy cattle diseases collectively cause over $65 billion in global losses each year with subclinical ketosis, clinical mastitis, and subclinical mastitis being the costliest (Rasmussen et al., 2024). These numbers highlight the need for effective herd health monitoring and management tools to mitigate disease-related losses.

Early detection of health issues is vital, but current monitoring approaches face technological and economic limitations. Detecting diseases through chemical measurement of specific biomarkers for each disease is labor-intensive, costly, and sometimes invasive (e.g., blood sampling), which restricts large-scale implementation, causes discussions from an ethical perspective, and is impractical for routine use on farm. Consequently, there is strong interest in affordable, high-throughput alternative methods that can leverage existing data streams to flag cows at risk of suboptimal health. In this context, Fourier-transform mid-infrared (**FT-MIR**) spectroscopy has emerged as a widely available tool. Milk recording organizations already use FT-MIR analysis to measure milk composition for millions of cows, providing rapid, noninvasive insight into each cow's physiology (Soyeurt et al., 2012; De Marchi et al., 2014).

Indeed, these FT-MIR spectra are used to predict an array of major and minor milk components but also health-related biomarkers such as indicators of energy balance (e.g., citrate, oleic acid), ketosis (e.g., milk and blood BHB, milk acetone, blood nonesterified fatty acids), or subclinical mastitis (lactoferrin, N-acetyl-β-D-glucosaminidase; Soyeurt et al., 2012; Grelet et al., 2016; Giannuzzi et al., 2023). Nevertheless, the accuracy of each individual FT-MIR models can vary widely with coefficients of determination from 0.3 to 0.8 (Grelet et al., 2021), partly due to low concentrations in milk, indirect predictions for concentration in blood, limited data on sick cows, and the skewed distributions of the concentrations in milk (Grelet et al., 2024). These constraints make it difficult to predict health conditions reliably via single biomarkers alone.

To extract more global and complementary health information from milk records, researchers have turned to multivariate data mining approaches such as clustering with the biomarkers (Grelet et al., 2019; Foldager et al., 2020). Franceschini et al. (2022) recently demonstrated that it is possible to uncover latent patterns related to cow health by clustering milk recording data (i.e., milk yield, SCC, and 27 FT-MIR–predicted traits) from over 100,000 primiparous Holstein cows. Their analysis identified 5 distinct clusters of records. Notably, one cluster was associated with metabolic disorder indicators and another with mastitis-related measures. Significant correlations were observed between the probabilities of the metabolic cluster and several reference measurements associated with energy balance and ketosis, including blood BHB (0.70), blood nonesterified fatty acids (0.67), blood glucose (−0.60), milk glucose-6-phosphate (−0.32), milk BHB (0.53), milk isocitrate (0.50), and milk urea (0.32). These novel cluster-derived phenotypes aggregate multiple signals and could serve as holistic health indicators for on-farm management, while being less sensitive to individual model inaccuracy. Hu et al. (2025) also showed that this metabolic disorder cluster information was relevant for genetic purposes.

Despite the promise of this unsupervised approach, important questions remain about its robustness and practical utility. The previous study was limited to first-lactation Holsteins, and it remains unclear whether similar health-related spectral patterns emerge in later lactations. Additionally, the stability (i.e., reproducibility) of the clusters found from different datasets has not been thoroughly examined while the divide-and-conquer approach includes randomness. Furthermore, using the clustering method in practice requires computing many intermediate predictor traits from FT-MIR, adding complexity to the workflow.

In the present study, we hypothesized that the metabolic cluster previously identified in primiparous Holstein cows is reproducible across lactations and can be predicted directly from FT-MIR spectra. In addition, the mastitis-related cluster was also assessed across lactations even though its strongest signal was SCC, which is not predicted from spectra. We aimed to address this hypothesis by evaluating the clustering approach across lactations and by testing the consistency and reproducibility of the results under repeated analyses. We also evaluated a new strategy to predict the cluster-defined phenotype directly from milk FT-MIR spectra, bypassing the need to predict multiple biomarkers, which could become a limitation when the prediction equations are not available to stakeholders. Although this direct prediction approach may reduce interpretability compared with biomarker-based approaches, it could enhance the transferability and implementation of the method. Ultimately, such an approach could facilitate large-scale monitoring of cow health and contribute to improved disease management and animal welfare.

Because one of the aims of this research is to compare the results of the previous research with primiparous cows detailed by Franceschini et al. (2022) to multiparous cows, the dataset and the general clustering algorithm are similar. However, additional methods are developed to assess robustness and compare the groups made by the clustering. All data treatments and analyses were performed using R software (version 4.3.2; https://www.r-project.org/). All data were collected as per standard routine by the Walloon Breeders Association (Elevéo, Awé groupe, Ciney, Belgium), which is accredited to perform such collection. The methodology deployed in this study is summarized in Figure 1, and the different steps discussed in this communication follow the structure of this workflow.

**Figure 1:**
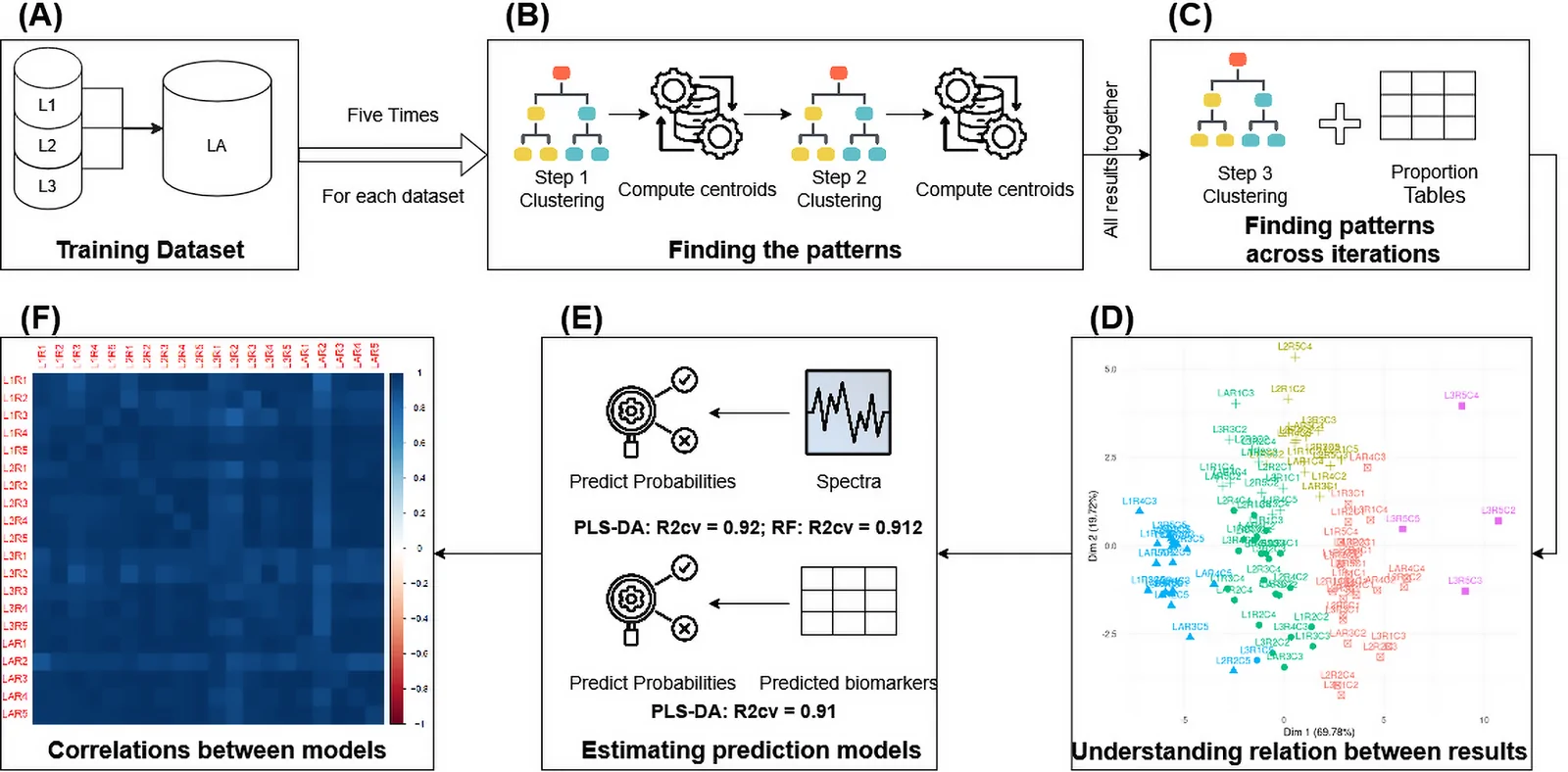
Methodology of the study: For each of the 4 datasets and 5 repetitions, data were clustered 2 times, respectively, for the divide-and-conquer approach and formal clustering. L1, L2, and L3 = dataset for lactation 1, 2, and 3, respectively; LA = dataset with all the data. A final clustering was performed to create the overall groups across iterations. The relation between clusters were studied and prediction models were estimated. Correlations between models were estimated to compare the results. Dim = dimension of the principal component analysis; R2cv = coefficient of determination in cross-validation; R = repetition; C = cluster.

Data from this study came from the milk recording routine in southern Belgium between 2012 and 2020. In total, there were 1,712,417 DHI records from 1,082 herds and 139,203 Holstein cows with DIM ranging between 5 and 365 d. The records were divided into 3 datasets based on the lactation number: 740,454 for first, 577,601 for second, and 394,362 for third lactation. The DHI records contained the milk yield, SCC, DIM, and FT-MIR spectrum. Those analyses were performed by the milk laboratory “Comité du Lait” (Battice, Belgium) on Foss Milkoscan FT6000, FT+, and FT7 standardized spectrometers. The spectral standardization method was described in Grelet et al. (2015, 2017). As advised by Grelet et al. (2021), spectra were preprocessed through a first derivative, and 212 wavelengths were selected.

The 27 FT-MIR–based biomarkers used in the study were the same as those presented in Franceschini et al. (2022) and predicted with the same equations. The 28th and 29th biomarkers were the milk yield and SCS. Compared with Franceschini et al. (2022), SCC was replaced by the SCS, to avoid the potential effect of the asymmetrical distribution, with this formula:

$$SCS=log_{2}\left(\frac{SCC}{100,000}\right)+3.$$

Descriptive statistics for all variables are available in the supplemental material (see Notes). The datasets were used both separately by lactation and all together to verify whether the developed method was applicable on multiparous cows. So, 4 datasets were used, one for each lactation number, and one more with all the data (Figure 1A). Moreover, the procedure was repeated 5 times for each dataset for a total of 20 separate analysis. This data splitting ensured that we could assess the reproducibility of the clustering approach within and between lactations while also evaluating the robustness of the identified clusters across iterations.

As developed in Franceschini et al. (2022), a 2-step hierarchical clustering method using the Euclidean distance had been performed. Indeed, it was not possible to use such a volume of data simultaneously because hierarchical clustering requires the computation of a distance matrix, which would require excessive memory allocation. Instead, the dataset was randomly split into 100 smaller datasets that were clustered separately. Then, the multiple hierarchical clustering were partitioned to obtain 600 groups of records for each. The means of each group called centroid were calculated to summarize the information. Then, a second hierarchical clustering took as input 60,000 centroids (i.e., 100 subsets × 600 centroids). Finally, we cut the final dendrogram to obtain 5 large groups of centroids as in Franceschini et al. (2022), and we computed the means for the different variables (Figure 1B). Those groups will be called “clusters” in the following text.

As the whole approach was repeated 5 times for each of the 4 datasets, we obtained 20 outputs of 5 clusters each, for a total of 100 clusters. To assess if these 100 clusters represented the same 5 clusters for each repetition, a last hierarchical clustering was performed. The dendrogram from the final hierarchical clustering algorithm was also divided into 5 groups called the overall group (Figure 1C).

The objective of this last clustering was only to validate that the metabolic disruption and mastitis clusters from the previous study were found in every repetition. If the signals from the clusters were strong and similar in every repetition, there should be 5 overall groups containing 20 similar clusters each. However, the result showed a different distribution with 26 in overall group 1, 14 in overall group 2, 36 in overall group 3, 20 in overall group 4, and 4 in overall group 5 (Table 1). So, the different partitions from the 20 repetitions were not robust. Only overall group 4 contains 20 clusters, one for each of the 5 repetitions across 4 lactation datasets.

**Table 1:** Assignment of the 100 clusters (5 clusters per repetition × 5 repetitions per lactation dataset × 4 lactation datasets) to the 5 overall groups^1^

Group	Lactation 1					Lactation 2					Lactation 3					All lactations					Total
	R1	R2	R3	R4	R5	R1	R2	R3	R4	R5	R1	R2	R3	R4	R5	R1	R2	R3	R4	R5	
1	1	1	1	1	2	1	3	1	1	1	3	1	1	1	0	1	1	1	3	1	26
2	1	0	0	1	1	2	0	1	1	2	0	0	1	1	0	1	0	1	0	1	14
3	2	3	3	2	1	1	1	2	2	1	1	3	2	2	0	2	3	2	1	2	36
**4**^2^	**1**	**1**	**1**	**1**	**1**	**1**	**1**	**1**	**1**	**1**	**1**	**1**	**1**	**1**	**1**	**1**	**1**	**1**	**1**	**1**	**20**
5	0	0	0	0	0	0	0	0	0	0	0	0	0	0	4	0	0	0	0	0	4

1 R = repetition.

2 Group 4 in bold is consistent across repetitions and lactations.

To identify whether this overall group 4 represented the metabolic disruption or the mastitis cluster found by Franceschini et al. (2022), we looked at a principal component analysis (Figure 1D) and the predictions. Most specifically, we looked at the cluster with the highest value of acetone and BHB in milk and the lowest value of energy balance (**EB**) in each repetition (Figure 2). Except for one repetition (EB in the first repetition of the third lactation dataset), overall group 4, which was in every repetition, was also the one with the highest value of acetone and BHB and the lowest value of EB. So, it should be associated with metabolic troubles.

**Figure 2:**
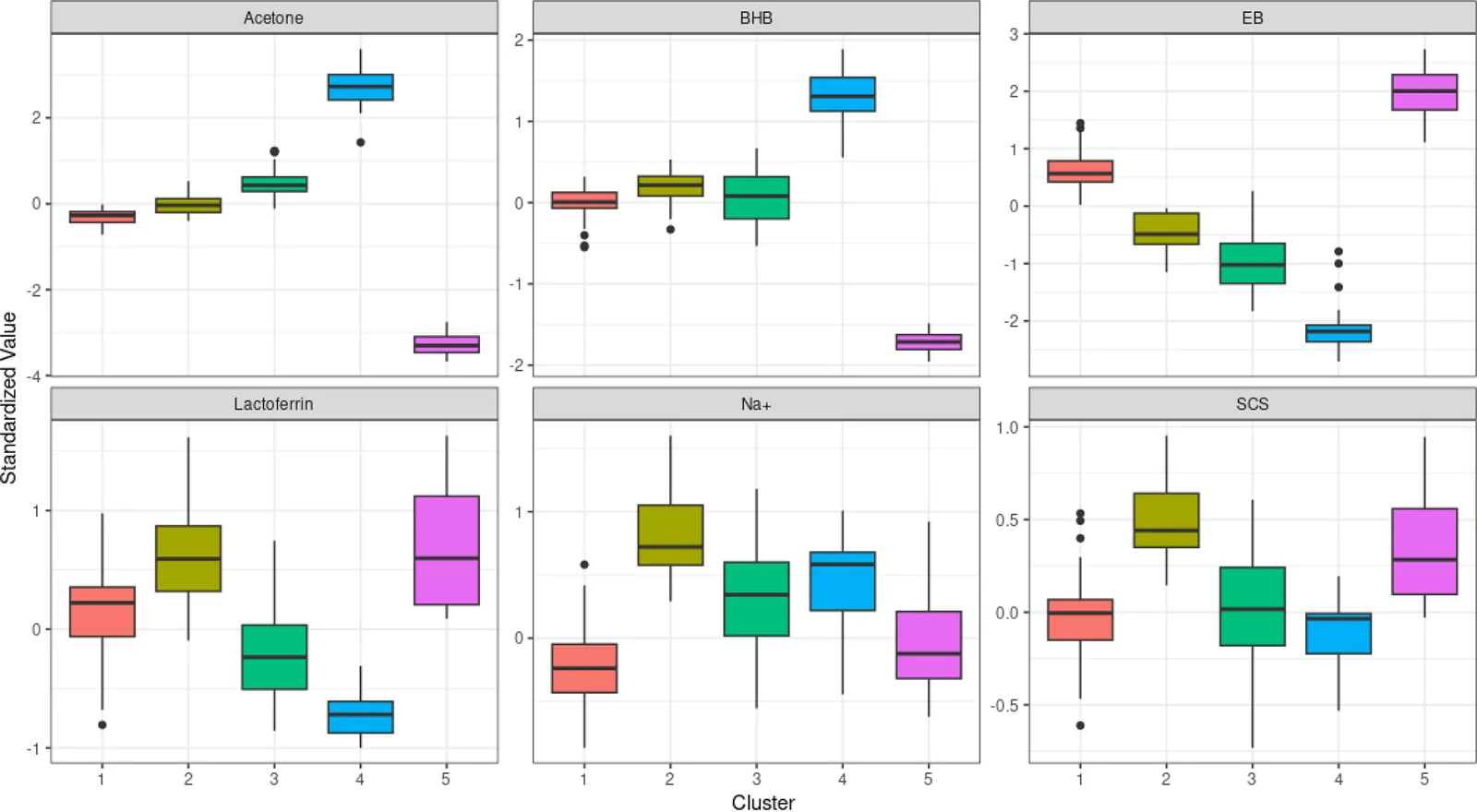
Boxplots of the distributions of the different clusters for biomarkers of interest. EB = energy balance. The line represents the median. The lower and upper edges of the box correspond to the first and third quartiles (the 25th and 75th percentiles). The whiskers represent 1.5 times the interquartile range. Observations beyond that range are considered as outliers.

A similar approach was used to find the cluster associated with mastitis, we looked at the highest value of SCS, lactoferrin, and Na^+^ (Figure 2). However, the results were not as consistent. Overall group 2 was associated with the highest values of the 3 variables in 7 out of the 20 repetitions. Even if we only considered SCS, overall group 2 is only found in 10 of the repetitions. So, the mastitis cluster was not as stable. As hypothesized previously, most of the signal found in Franceschini et al. (2022) for mastitis might come from the highly skewed distribution of the SCC, which might give more weight to this variable instead of the FT-MIR predictions. When the SCS was used instead of SCC, the signal of the mastitis cluster was weaker, and we could not identify the pattern across lactations and iterations. For all the reasons mentioned, the cluster related to mastitis was not considered for the repetition analysis.

To further evaluate whether the information in the metabolic troubles cluster was identical across iterations, we created 20 prediction models, one for each of the 4 datasets and each of the repetitions, using the 29 predicted features but also 20 models using directly the FT-MIR spectra (Figure 1E). Indeed, using the spectra directly allows a faster and easier estimation of the cluster values and probabilities.

Each model was estimated with data used for the clustering approach. So, a model for a cluster found from the first-lactation dataset was estimated using a cross-validation on the first-lactation dataset. The idea was that if the clusters were similar, the predictions from the different models should be highly correlated. The 29 features were used to predict the cluster using partial least square discriminant analysis (**PLS-DA**) as in Franceschini et al. (2022). The same approach was applied to the FT-MIR spectra to develop an equation. The output for each observation was the probability to belong to the metabolic trouble cluster. To ensure a class balance between metabolic troubles and healthy observations, datasets were downsampled by sampling the same number of observations for each. Then a 10-fold cross-validation was applied to define the best number of latent variables with the train function and the *oneSE* approach from *caret* to estimate the model quality (Kuhn, 2008; Breiman et al., 2017).

The numbers of latent variables required were between 1 and 3 for models using predictions and between 10 and 19 for models using FT-MIR spectra. The retained latent variables explained between 31.4% and 48.2% of the variance in the predictor matrix for models using predictions and between 76.4% and 92.7% for models using FT-MIR spectra. The accuracies in cross-validation ranged between 0.79 and 0.93 using predictions and between 0.76 and 0.93 using FT-MIR spectra. So, the model performances were quite different between repetitions, but those performances were not comparable because the datasets were different.

After the models trained on predictions and spectra were estimated, we applied those models to the full dataset LA and computed the Pearson correlations. Those correlations are shown in the final heatmap of Figure 1F. This methodology allowed us to compare the results of the models and see whether they predict the same information. Overall, the weakest correlation was 0.82 and the strongest is 1.00, so the predictions for a metabolic cluster were mostly identical across lactations and repetitions. Correlations between models trained from predictions or FT-MIR spectra in the same repetitions (i.e., with the same definition of the metabolic trouble cluster) were between 0.93 and 0.95, also containing the same information. The model having the best average correlation with all the others was trained using the full dataset LA and the first repetition. This would suggest that the signal for metabolic issues was strong enough in the spectra so that the pattern could be identified in every dataset across lactation and every repetition.

The PLS-DA prediction model using spectra we kept was estimated with the full dataset LA and the first repetition. The accuracy in cross-validation for this model is 0.920 ± 0.002. A random forest (**RF**) model was also estimated as it might capture the nonlinearity while also offering a probability-like output by looking at proportion of trees voting for one cluster (Franceschini et al., 2025). This algorithm was only used on the LA results because of the computational burden of such estimation for 1,712,417 records and 212 spectral wavelengths. A 10-fold cross-validation was used to optimize the number of variables selected at each split and the minimum node size. We used Gini as the split rule; the number of trees was 500 and we limited the maximum depth to 15 to avoid a model being too complex. The best RF uses 50 variables at each split and a minimum node size of 5. The average accuracy in cross-validation of the model is 0.912 ± 0.001. A paired *t*-test was applied by pairing the differences in performance between both methods for each of the 10-folds to determine whether this difference in accuracy is significant, and the result is highly significant (*P* < 0.001) even though the average difference is 0.008. So, the PLS-DA model was slightly better than the RF model. This result could be explained by the maximum depth added to simplify the RF model or by the absence of nonlinearity. However, probability outputs by both algorithms were distributed quite differently with a normal distribution around 0.5 for the PLS-DA and a bimodal distribution for the RF. This difference, combined with the computational burden, could justify the use of both algorithms to fine-tune a detection threshold relevant for the farmer.

Several limitations should nevertheless be considered. First, although the metabolic cluster was reproducible across lactations and repeated iterations, the robustness of cluster assignment under changing population structures, breeds, or management systems outside southern Belgium still requires further investigation. Additional methodologies, such as introducing perturbations in the datasets and evaluating the agreement of the resulting cluster assignments, could further strengthen the reproducibility assessment. Second, the hierarchical clustering strategy relied on a divide-and-conquer approach because the computation and storage of large distance matrices generated substantial computational and memory requirements, which could potentially be reduced using alternative unsupervised learning approaches. Finally, the use of Euclidean distance and distance-based clustering approaches may be influenced by nonlinear relationships and correlated predictors, potentially affecting the relative importance attributed to some variables.

In conclusion, this research proposed a way to assess the generalizability across lactations and the repeatability of the approach developed while also presenting an equation to predict the metabolic trouble cluster directly from the spectra for better transferability. Those results showed that only one of the 2 clusters previously detected is relevant. Indeed, the metabolic trouble cluster is consistent across lactations and repetitions, which highlight its strong signal in FT-MIR. This cluster could be used directly in the routine to assess metabolic issues on farm or to screen for future sampling. The next step would be to validate using this on-farm implementation and to assess the cluster generalizability to other countries and breeds. However, this validation requires a framework where the information is quickly streamlined to the farmer and feedback is collected. The direct prediction from FT-MIR is a first step in this direction.
